# Isolation of Pancreatic Progenitor Cells with the Surface Marker of Hematopoietic Stem Cells

**DOI:** 10.1155/2012/948683

**Published:** 2012-12-20

**Authors:** Fengxia Ma, Fang Chen, Ying Chi, Shaoguang Yang, Shihong Lu, Zhongchao Han

**Affiliations:** State Key Laboratory of Experimental Hematology, Institute of Hematology and Hospital of Blood Disease, Chinese Academy of Medical Sciences, Tianjin 300020, China

## Abstract

To isolate pancreatic progenitor cells with the surface markers of hematopoietic stem cells, the expression of stem cell antigen (Sca-1) and c-Kit and the coexpression of them with pancreatic duodenal homeobox-1 (PDX-1), neurogenin 3 (Ngn3), and insulin were examined in murine embryonic pancreas. Then different pancreatic cell subpopulations were isolated by magnet-activated cell sorting. Isolated cells were cultured overnight in hanging drops. When cells formed spheres, they were laid on floating filters at the air/medium interface. With this new culture system, pancreatic progenitor cells were induced to differentiate to endocrine and exocrine cells. It was shown that c-Kit and Sca-1 were expressed differently in embryonic pancreas at 12.5, 15.5, and 17.5 days of gestation. The expression of c-Kit and Sca-1 was the highest at 15.5 days of gestation. c-Kit rather than Sca-1 coexpressed with PDX-1, Ngn3, and insulin. Cells differentiated from c-Kit-positive cells contained more insulin-producing cells and secreted more insulin in response to glucose stimulation than that from c-Kit-negative cells. These results suggested that c-Kit could be used to isolate pancreatic progenitor cells and our new culture system permitted pancreatic progenitor cells to differentiate to mature endocrine cells.

## 1. Introduction

Identification and isolation of pancreatic progenitor cells generate much interest due not only to their developmental importance but also to their therapeutic potential for diabetes [1]. However, the prospective isolation of pancreatic progenitor cells is difficult because of the lack of specific markers and a cell culture system to determine their capacity to differentiate [2]. Pancreas duodenum homeobox-1 (PDX-1) plays an important role in pancreatic development and is a marker of progenitor cells which can differentiated to endocrine and exocrine cells [3]. Neurogenin 3 (Ngn3) is a marker of progenitor cells which can differentiated to endocrine cells [4]. However, PDX-1 and Ngn3 are unsuitable for isolating progenitor cells by cell sorting, because they are nuclear transcription factors rather than surface markers. Hematopoietic stem cells (HSC) were successfully isolated from bone marrow using surface markers such as CD133, stem cell antigen-1 (Sca-1), and c-Kit [5, 6]. Recently, it has been shown that CD133 are used to isolate pancreatic progenitor cells [7].

Sca-1 and c-Kit also were expressed in pancreas. The expression of c-Kit in the early rat pancreas was first reported by Scharfmann's group [8]. Later, they identified that c-Kit was a potential marker for islet progenitor cells [9]. Wang's group reported that c-Kit was expressed in endocrine regions of postnatal pancreas [10]. They also showed that isolated c-Kit-positive cells can be expanded in vitro and give rise to new *β*-cells that secrete insulin in a glucose-responsive fashion [11]. Recently, they found that c-Kit may be a marker for human pancreatic islet progenitor cells and involved in mediating early *β*-cell differentiation and survival [12]. Outside the hematopoietic system, Sca-1 was also expressed by stem/progenitor in a wide variety of tissues and organs such heart, liver, and prostate [13]. Recently, it was shown that islet and ductal cells in adult pancreas expressed Sca-1 [14]. The present study will explore if c-Kit and Sca-1 are suitable as surface markers to isolate murine pancreatic progenitor cells.

## 2. Materials and Methods

### 2.1. Reagents

Antibodies of pancreatic markers for immunohistochemistry were obtained from Santa Cruz Biotechnology (Santa Cruz, CA). Antibodies of pancreatic markers for flow cytometry were obtained from R&D Systems (Minneapolis, USA). Unconjugated and phycoerythrin (PE) or FITC conjugated Sca-1 and c-Kit antibody were purchased from BD (Franklin Lakes, NJ). Insulin ELISA kit was purchased from Mercodia (Uppsala, Sweden).

### 2.2. Animals

Pregnant Swiss mice aged 6–8 weeks were purchased from Vital River Experimental Animals Company (Beijing, China). The morning postcoitum was designated as embryonic day (E) 0.5. At E12.5, E15.5, and E17.5 days of gestation, pregnant mice were killed by cervical dislocation, according to the guidelines of the Chinese Animal Care Committee's guidelines. Murine embryos were removed from the uterus. The digestive tracts were isolated and the dorsal pancreatic rudiments were dissected.

### 2.3. Dissociation of Pancreatic Rudiment

Dorsal pancreatic rudiments were digested by collagenase (Sigma) at 37°C for 15 min. After digestion, cells were washed two times with RPMI 1640 and mechanically dispersed through syringe needle. Dissociated cells were filtered by sterile nylon filter and single cells suspension was prepared. 

### 2.4. Flow Cytometric and Immunofluorescent Analysis

For intracellular PDX-1, Ngn3, and insulin dectection, cells were prepenetrated with 0.1% Triton X-100 for 15 min at 37°C before being labeled with antibody. For Sca-1 and c-Kit dectection, cells were incubated for 30 min at 4°C with PE conjugated Sca-1 or c-Kit antibody, isotype-matched antibodies served as controls. Cells were then fixed in 1% polyformaldehyde and quantitatively analyzed by flow cytometry. After flow cytometry analysis, cells were put on a slide with cytospin and observed under fluorescence microscopy.

### 2.5. RNA Extraction and PCR

Total RNA was extracted with trizol (Invitrogen). cDNA synthesis was performed by using MLV RT (Promenga) for 2 h at 37°C in the presence of oligo-dT primer. Specific primers were used to identify and amplify Sca-1 and c-Kit. The PCR-amplified samples were visualized on 1% agarose gels using ethidium bromide.

Real-time PCR was performed using SYBR Green Master Mix (Applied Biosystems) on 7500 Real-time PCR system. The comparative method of relative quantification (2^−ΔΔ*ct*^) was used to calculate expression levels of each target gene, normalized to GAPDH. The data were presented as fold changes in gene expression.

### 2.6. Immunohistochemistry and Quantification

Tissues were fixed in formalin, preembedded in 4% low gelling temperature agarose, and embedded in paraffin. Sections (4 *μ*m thick) were collected and processed for immunohistochemistry. The first antibodies were diluted to 1/1000. The fluorescent secondary antibodies were diluted to 1/200. Nuclei were stained in blue using DAPI.

To quantify the surface area of staining, all sections of each sphere were digitized. One of two consecutive sections was analysed by immunohistochemistry in order to avoid counting the same cell twice. The surface of staining was quantified using ImageJ.

### 2.7. Isolation of c-Kit-Positive and Negative Cells

To isolate c-Kit-positive and negative cells, pancreatic cells were incubated with PE conjugated c-Kit antibody for 30 min at 4°C. After wash, cells were incubated with anti-PE microbeads (Miltenyl Biotec). After passing through a magnetic column twice, c-Kit-positive and-negative cells were respectively collected.

### 2.8. Pancreatic Cell Culture after Isolation (New Culture System)

Isolated c-Kit-positive and negative cells were suspended with RPMI 1640 at the density of 5000 cells/*μ*l. 20 *μ*l cell suspension was plated in Terasaki dish, then the dish was overturned. Because of gravity, cell suspension turned into hanging drop. After overnight in hanging drops, cells gathered together and formed spheres. Pancreatic spheres were cultured for 6 days on 0.45 *μ*m floating filters at the air/medium interface in Petri dishes, containing RPMI 1640 supplemented with 10% fetal bovine serum, penicillin, streptomycin, HEPES, non-essential amino acids, L-glutamine. Cultures were maintained at 37°C in humidified 95% air/5% CO_2_.

### 2.9. Insulin Secretion

Pancreatic spheres were placed in a 96-well plate, one sphere per well, and washed three times with Krebs-Ringer bicarbonate (KRB) buffer with 0.5% BSA. Then spheres were preincubated in 150 *μ*l KRB buffer with 0.5% BSA for 60 min, then incubated in 150 *μ*l KRB buffer containing 5 mM glucose or 25 mM glucose for 120 min at 37°C. The reaction was stopped by placing the plates on ice for 5 min. KRB buffer was collected and used to measure the levels of insulin released with an insulin ELISA kit.

### 2.10. Statistical Analysis

Data were expressed as mean ± SD. Differences between group means were determined by Student's *t*-test. *P* < 0.05 was considered statistical significance.

## 3. Results

### 3.1. The Expression of Sca-1 and c-Kit in Embryonic Pancreas

**Figure 1:**
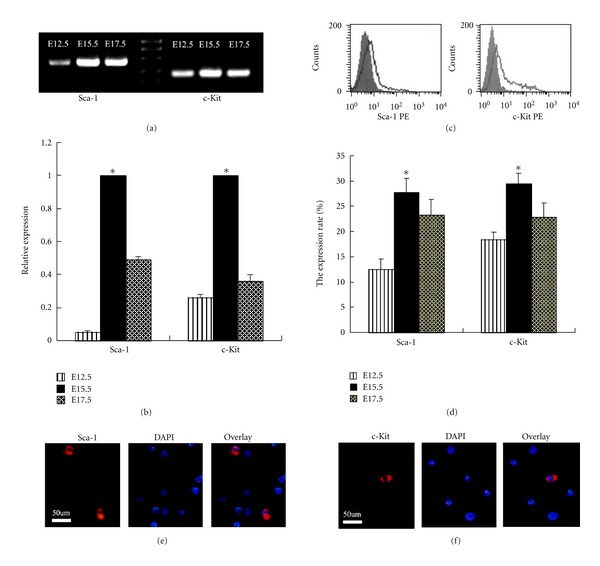
The expression of Sca-1 and c-Kit in embryonic pancreas. Representative picture of agarose gel electrophoresis (a). The quantitive analysis of Sca-1 and c-Kit mRNA expression by real-time PCR (b). Representative flow cytometric results of Sca-1 and c-Kit expression at E15.5 (c). The quantitive analysis of Sca-1 and c-Kit protein expression (d). Immunofluorescent analysis of Sca-1 and c-Kit localization (e, f). *P < 0.05 versus E12.5 and E15.5.

PCR analysis showed that Sca-1and c-Kit were expressed in embryonic pancreas of E12.5, E15.5, and E17.5 (Figure 1(a)). In order to quantitate the expression, real-time PCR was performed. It was shown that the expression of Sca-1 and c-Kit at E15.5 was higher than that at E17.5 and E12.5 (Figure 1(b)). Flow cytometric analysis showed that the protein expression of Sca-1 and c-Kit at E15.5 was also higher than that at E17.5 and E12.5 (Figures 1(c) and 1(d)). Immunofluorescent analysis also showed that Sca-1 and c-Kit were localized in the membrane rather than nucleus of pancreatic cells (Figures 1(e) and 1(f)).

### 3.2. The Coexpression of Sca-1 and c-Kit with Pancreatic Markers

**Figure 2:**
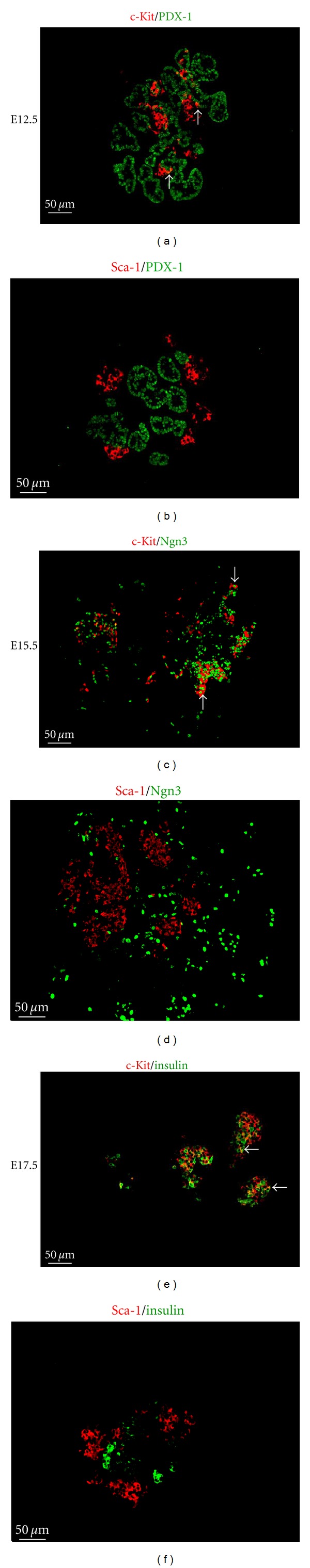
The coexpression of Sca-1 and c-Kit with PDX-1, Ngn3, and insulin in embryonic pancreas at E12.5, E15.5, and E17.5 by immuno-histochemistry. White arrow marked c-Kit-positive cells coexpressed with PDX-1, Ngn3, and insulin.

**Figure 3:**
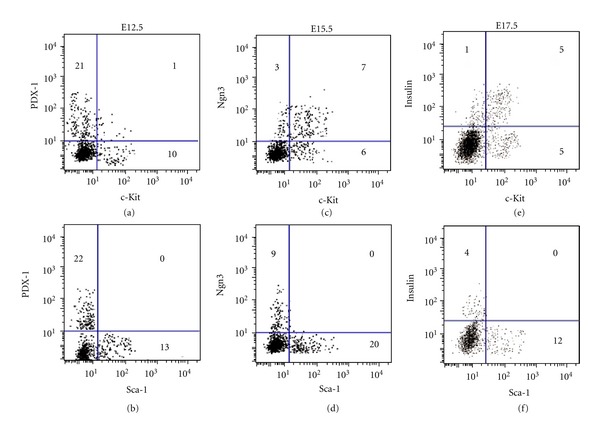
The coexpression of Sca-1 and c-Kit with PDX-1, Ngn3, and insulin in embryonic pancreas at E12.5, E15.5, and E17.5 by flow cytometry.

At E12.5, 25 ± 6% embryonic pancreatic cells expressed PDX-1. Furthermore, 8 ± 3% c-Kit-positive cells expressed PDX-1 (Figures 2 and 3(a)). However, PDX-1 and Sca-1 were not coexpressed (Figures 2 and 3(b)). In embryonic pancreas at E15.5, 62 ± 9% c-Kit-positive cells coexpressed with Ngn3 (Figures 2 and 3(c)), however, no Sca-1 positive cells coexpressed with Ngn3 (Figures 2 and 3(d)). In embryonic pancreas at E17.5, 53 ± 8% c-Kit-positive cells coexpressed with insulin (Figures 2 and 3(e)); however, no Sca-1 positive cells coexpressed with insulin (Figures 2 and 3(f)). Different from HSC, In embryonic pancreas of E12.5, E15.5, E17.5, Sca-1, and c-Kit did not coexpress with each other (data not shown).

### 3.3. The Validation of Our New Culture System

**Figure 4:**
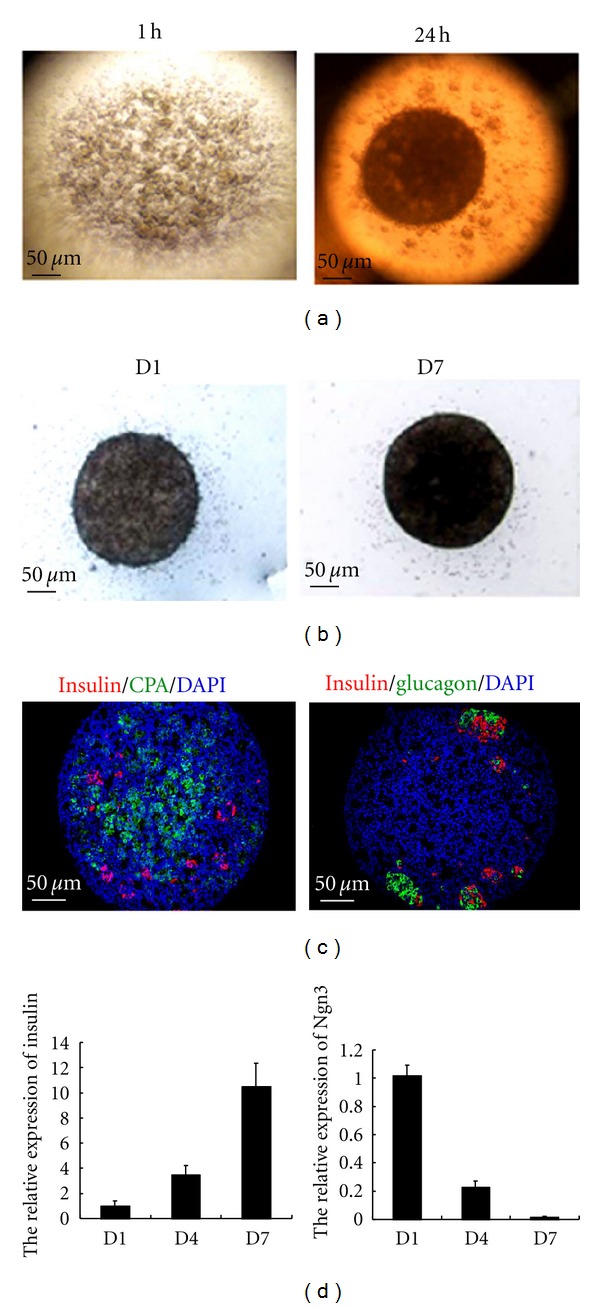
The characteristics of our new culture system. (a) The photo of pancreatic cells cultured in inverted dish for 1 h and 24 h. (b) The morphology of spheres cultured for 1 and 7 days at the air-medium interface. (c) Immunohistological analysis of insulin, glucagon, and CPA staining in spheres cultured for 7 days. (d) The expressional dynamics of Ngn3 and insulin mRNA in spheres cultured for 1, 4, 7 days.

100,000 unsorted pancreatic cells at E15.5 were cultured in inverted Terasaki dishes. Overnight, cells formed spheres because of gravity (Figure 4(a)). The density of spheres increased after culture for 6 days on floating filter. The reason was that pancreatic progenitor cells gradually differentiated into endocrine and exocrine cells which consisted of a large amount of secretory particles (Figure 4(b)). Insulin, glucagon were expressed in spheres cultured for 7 days. Insulin^+^ and glucagon^+^ cells gathered together and formed the structures like pancreatic islets. Carboxypeptidase A (CPA) was also expressed in spheres cultured for 7 days. (Figure 4(c)). The expressional dynamics of pancreatic markers in cultured spheres was examined by PCR. The expression of Ngn3 gradually decreased and the expression of insulin increased during culture (Figure 4(d)).

### 3.4. The Characteristics of c-Kit-Positive and-Negative Embryonic Pancreatic Cells

**Figure 5:**
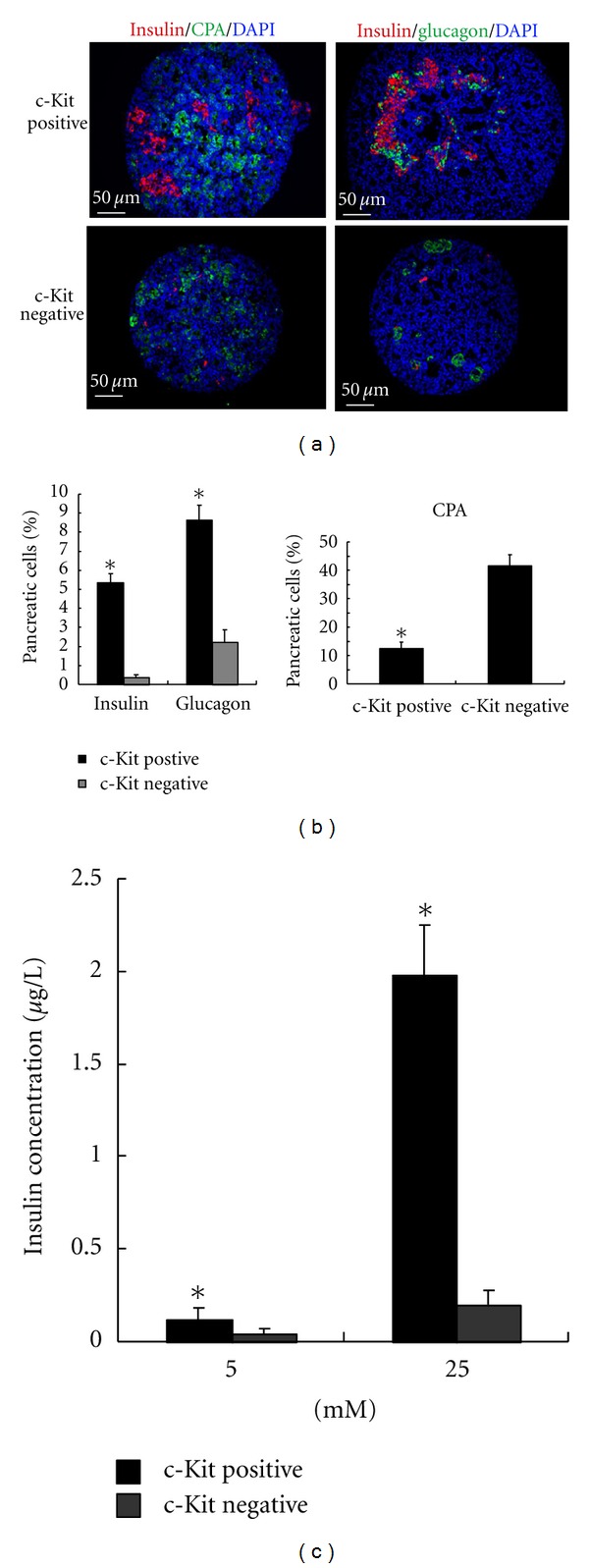
The comparison of spheres differentiated from c-Kit-positive and-negative cells. (a) Immunohistological staining of insulin, glucagon, and CPA in spheres. (b) Quantitative analysis of insulin and glucagon expression in spheres. *P < 0.05 versus c-Kit-negative cells. (c) Quantitative analysis of CPA expression in spheres. *P < 0.05 versus c-Kit-negative cells. (d) Insulin secretion in response to 5 mM and 25 mM glucose in spheres. *P < 0.05 versus c-Kit-negative cells.

The percentage of c-Kit-positive in embryonic pancreas at E15.5 was 24% ±1.6%. After sorting, the purity of c-Kit-positive was 90% ±4%. c-Kit-positive and negative cells both differentiated into endocrine and exocrine cells cultured in our culture system. The volume of spheres developed from c-Kit-positive cells was greater than that of spheres developed from c-Kit-negative cells (Figure 5(a)). The percentage of insulin and glucagon of spheres developed from c-Kit-positive cells was more than that of spheres developed from c-Kit-negative cells (Figures 5(a) and 5(b)). However, The percentage of CPA of spheres developed from c-Kit-positive cells was less than that of spheres developed from c-Kit-negative cells (Figures 5(a) and 5(c)). In order to know if insulin-expressing cells in spheres were functional, insulin secretion in response to glucose was further explored. 0.20 ± 0.08 and 1.98 ± 0.27 *μ*g/L insulin was, respectively, released by spheres developed form c-Kit-positive cells in response to 5 mM and 25 mM glucose (Figure 5(d)). However, 0.05 ± 0.02 and 0.1 ± 0.06 *μ*g/L insulin was, respectively, released by spheres developed from c-Kit-negative cells in response to 5 mM and 25 mM glucose.

## 4. Discussion

In the present study, we showed that c-Kit and Sca-1 were expressed in a dynamic fashion in the developing murine embryonic pancreas. Furthermore, we demonstrated that c-Kit rather than Sca-1 was a marker of pancreatic progenitor cells with our new culture system. Although previous study demonstrated the coexpression of c-Kit with insulin and glucagon [9–11], to our knowledge, it was the first time to study the coexpression of c-Kit and Sca-1 with PDX-1 and Ngn3. At E12.5, few c-Kit-positive cells expressed PDX-1. It was interesting to study the characteristics of these double positive cells and their role in pancreatic development. However, these double positive cells were difficult to isolate and too few to be used for cell therapy without expansion. At E15.5, high percentage of c-Kit-positive cells expressed Ngn3. However, c-Kit-positive cells wasn't pure endocrine progenitor cells. To isolate highly pure endocrine progenitor cells, it is essential to combine c-Kit with other surface markers. c-Kit-expressing cells were also purified from epithelial monolayers derived from postnatal rat pancreatic islets in Wang's group [11]. They also demonstrated that c-Kit-positive cells potentially contribute to the process of new islet cluster formation. Thus, the present study further showed direct evidence that c-Kit could be used as a surface marker of endocrine progenitor cells, which would be critical for developing new islet cell-based therapies. Although we showed that cells differentiated from c-Kit-positive cells secreted more insulin than cells differentiated from c-Kit-negative cells. If blood glucose was decreased in hyperglycaemic mice after transplantation of c-Kit-positive cells needed to be further investigated.

In previous studies, it was shown that Sca-1 was expressed in nonhematopoietic progenitor cells, such as mammary gland epithelial progenitor cells, lung epithelial—specific progenitors and hepatic progenitor cells [15–17]. Seaberg et al. showed that 9% islet cells and 15% ductal cells of adult pancreas were Sca-1 positive. However, these Sca-1 positive cells did not formed pancreatic colonies [14]. Our results were consistent with Seaberg's result. Although Sca-1 wasn't the marker of pancreatic progenitor cells, the role of Sca-1 in embryonic pancreas needed to be further investigated. 

In the present study, we developed a new cell culture system to determine the capacity to differentiate of pancreatic cells. Pancreatic cells were cultured in hanging drop and formed spheres, which facilitated pancreatic cells to contact with each other as well as pancreas in vivo. We previously showed that this culture system mimicked pancreatic development in vivo and promoted the differentiation of endocrine cells [18]. More importantly, endocrine cells, especially insulin-producing cells, were able to be differentiated in spheres without any inducible factors. Furthermore, the kinetics of pancreatic marker expression in cultured spheres was coincident with that of pancreas in vivo. Our results demonstrated that our culture system was suitable for inducing pancreatic progenitor cells to differentiate. In addition to pancreatic progenitor cells, our culture system may be used to culture other type of stem/progenitor cells.

## Abbreviations

Sca-1: Stem cell antigen
Ngn3: Neurogenin 3
PDX-1: Pancreas duodenum homeobox-1
HSC: Hematopoietic stem cells
CPA: Carboxypeptidase A
E12.5: Embryonic day 12.5
PE: Phycoerythrin.
